# Increased Leg Strength After Concurrent Aerobic and Resistance Exercise Training in Older Adults Is Augmented by a Whole Food-Based High Protein Diet Intervention

**DOI:** 10.3389/fspor.2021.653962

**Published:** 2021-03-26

**Authors:** James F. Timmons, Michelle Hone, Karl E. Cogan, Orlaith Duffy, Brendan Egan

**Affiliations:** ^1^Institute for Sport and Health, School of Public Health, Physiotherapy and Sports Science, University College Dublin, Dublin, Ireland; ^2^School of Health and Human Performance, Dublin City University, Dublin, Ireland; ^3^Medfit Proactive Healthcare, Blackrock Co., Dublin, Ireland; ^4^National Institute for Cellular Biotechnology, Dublin City University, Dublin, Ireland; ^5^Florida Institute for Human and Machine Cognition, Pensacola, FL, United States

**Keywords:** body composition, combined training, nutrition, physical function, lean body mass (LBM)

## Abstract

Most studies in older adults have utilized powdered protein supplements or oral nutrition solutions as a source of additional dietary protein, but whole foods may provide a greater anabolic stimulus than protein isolated from food matrices. Therefore, the present study investigated a concurrent aerobic and resistance exercise training program in older adults, in the absence or presence of a high protein whole food-based dietary intervention, for effects on strength, physical function, and body composition. Community-dwelling older adults (*n* = 56; M/F, 28/28; age, 69.3 ± 4.0 years; BMI, 26.6 ± 3.7 kg m^−2^) participated in a 12-week intervention after randomization to either nutrition only (NUTR; *n* = 16), exercise only (EX, *n* = 19), or nutrition plus exercise (NUTR + EX, *n* = 21) groups. NUTR and NUTR + EX followed a dietary intervention targeting an increase in protein-rich meals at breakfast, lunch, and dinner. Exercise training in EX and NUTR + EX consisted of 24 min sessions of concurrent aerobic and resistance exercise performed three times per week. Daily protein intake increased in NUTR and NUTR + EX, but not EX. The increase in 1RM leg press strength was greater (Interaction effect, *P* = 0.012) in NUTR + EX [29.6 (18.1, 41.0) kg] than increases observed in NUTR [11.1 (−1.3, 23.6) kg] and EX [12.3 (0.9, 23.8) kg]. The increase in 1RM chest press strength was greater (interaction effect, *P* = 0.031) in NUTR + EX [6.3 (4.0, 8.6) kg] than the increase observed in NUTR [2.9 (0.3, 5.5) kg], but not EX [6.3 (3.9, 8.7) kg]. Hand-grip strength and sit-to-stand performance were each improved in all three groups, with no differences observed between groups (interaction effect, *P* = 0.382 and *P* = 0.671, respectively). An increase in percentage body fat was observed in NUTR, but not in EX or NUTR + EX (interaction effect, *P* = 0.018). No between-group differences were observed for change in lean body mass (interaction effect, *P* = 0.402). Concurrent aerobic and resistance exercise training improves strength and physical function in older adults, but combining this training with an increase in daily protein intake through whole foods may be advantageous to increase lower limb strength.

## Introduction

Age-related declines in skeletal muscle strength and physical function are a major threat to healthy aging by increasing the risks of adverse outcomes such as falls and fractures, frailty, loss of independence, and reduced quality of life (Wolfe, 2006; Cruz-Jentoft et al., 2019). These declines are exacerbated by the loss of skeletal muscle mass, and when declines in physical function and muscle mass are advanced, this results in the diagnosis of sarcopenia (Cruz-Jentoft et al., 2019). Beneficial effects of exercise training and/or high protein intake (>1.2 g kg^−1^ d^−1^) for older adults are evident in both epidemiological (McLean et al., 2016; Stamatakis et al., 2018) and intervention studies (Norton et al., 2016; Timmons et al., 2018) and form the basis of recommendations for maintaining skeletal muscle health in older adults (Chodzko-Zajko et al., 2009; Deutz et al., 2014; Bauer et al., 2019).

Interest in the “optimal” approach to lifestyle intervention in older adults at risk for functional decline have typically centered on combined resistance exercise and nutrition co-interventions (Liao et al., 2017; Ten Haaf et al., 2018; Labata-Lezaun et al., 2020). Concurrent aerobic and resistance exercise training, however, provides benefits to both aerobic fitness and strength-based outcomes (Karavirta et al., 2011), and even when time-matched tends to provide the same or better benefits compared to either mode alone (Wood et al., 2001; Timmons et al., 2018). However, little research has investigated strength, physical function, or body composition outcomes in older adults after undertaking concurrent aerobic and resistance exercise training when this training has been undertaken combined with a dietary intervention to increase daily protein intake.

Moreover, many studies to date have utilized powdered protein supplements or oral nutrition solutions as the source of additional dietary protein (Liao et al., 2017; Ten Haaf et al., 2018; Labata-Lezaun et al., 2020), but accumulating evidence suggests that whole foods may provide a greater anabolic stimulus than protein sources isolated from traditional food matrices (Elliot et al., 2006; Burd et al., 2015; van Vliet et al., 2017; Abou Sawan et al., 2018). Consequently, recent reviews have proposed the need for protein-based dietary interventions focusing on whole food sources for the provision of additional dietary protein, at least in community-dwelling older adults, given this potentially additive anabolic effect (Burd et al., 2019; Marshall et al., 2020). To maximize the anabolic effect of feeding throughout the day in older adults, it is also suggested on a per meal basis to include ≥2.5 g of the amino acid leucine within a protein dose ≥0.4 g kg^−1^, and for protein intake to follow an “even” distribution throughout the day (Traylor et al., 2019). Effects of such a pattern of intake from exclusively from whole food sources, either alone or in combination with exercise training, remain to be investigated.

Therefore, the present study investigated a concurrent aerobic and resistance exercise training program in older adults, in the absence or presence of a high protein whole food-based dietary intervention, for effects on strength, physical function, and body composition. The primary outcome under investigation was change in 1RM leg strength in response to intervention compared between groups. Leg strength was chosen as the primary outcome because of the observation of the age-related declines in muscle mass, muscle strength, and power being greater for the lower compared to upper limbs (Frontera et al., 1985; Lynch et al., 1985), and role of declining lower limb strength in the etiology of sarcopenia (Cruz-Jentoft et al., 2019). Secondary outcomes included changes in other measures of strength, physical function, body mass, and composition assessed both within and between groups. We hypothesized that this dietary intervention would augment exercise training-induced outcomes for leg strength and LBM compared to either exercise training or a high protein diet alone.

## Materials and Methods

### Experimental Design and Participants

A randomized trial using a parallel group, pre-post design, and comprising a 12-week intervention investigated the separate and combined effects of high protein diet and concurrent aerobic and resistance exercise training performed in men and women aged ≥ 65 years. All experimental procedures were approved by the University College Dublin Research Ethics Committee (permit: LS-17-22-Timmons-Egan) in accordance with the *Declaration of Helsinki*. Participants provided written informed consent prior to participation. Recruitment was primarily through the University College Dublin Alumni newsletter seeking men and women aged ≥ 65 years who were medically stable (Greig et al., 1994), community-dwelling, independent, fully mobile, and capable of completing the proposed intervention. Participants were excluded if they reported a history of myocardial infarction, cardiac illness, vascular disease, uncontrolled metabolic disease, stroke, or major systemic disease; or if already engaging in two or more structured exercise sessions per week.

An *a priori* sample size calculation (G^*^Power v3.1) required a sample size of 63 participants based on a three-group design (*n* = 21 per group) assuming to detect an effect size *f* of 0.2 [partial eta squared ($\eta_{\text{p}}^{2}$) = 0.04; “small”] for a given parameter at a Type I error rate (α) of 0.05 and a power (1–β) of 0.8. Upon entry to the study, participants (*n* = 63) were randomly assigned to one of three groups: nutrition only group (NUTR), concurrent aerobic and resistance exercise training only (EX), nutrition and concurrent aerobic and resistance exercise training (NUTR+EX) (CONSORT flow chart as Supplementary Figure 1). Assignment to the groups was performed by an independent researcher using random number generation and included stratified randomization by sex. Five participants from NUTR were lost to follow-up or discontinued the intervention, and two participants dropped out of EX due to inability to maintain the training frequency, leaving a final *n* size of 56 (NUTR, *n* = 16; EX, *n* = 19; NUTR+EX, *n* = 21; Table 1 and Supplementary Figure 1). Strength, physical function, and body composition were assessed before (PRE) and after (POST) 12 weeks of intervention. The POST assessment took place 48–96 h after the last training session for the EX and NUTR+EX groups.

**Table 1:** Participant characteristics at baseline (PRE).

	**NUTR (*n* = 16)** **mean ± SD**	**EX (*n* = 19)** **mean ± SD**	**NUTR+EX (*n* = 21)** **mean ± SD**	**ALL (*n* = 56)** **mean ± SD**	***P* value** **ANOVA**
M/F (*n/n*)	8/8	9/10	11/10	28/28	
Age (years)	69.3 ± 3.4	68.8 ± 3.8	69.7 ± 4.6	69.3 ± 4.0	0.769
**Anthropometry**					
Height (m)	1.69 ± 0.10	1.68 ± 0.10	1.69 ± 0.09	1.68 ± 0.09	0.933
Body mass (kg)	79.0 ± 8.8	72.5 ± 11.6	75.1 ± 13.0	75.3 ± 11.5	0.255
BMI (kg m^−2^)	28.0 ± 4.4	25.8 ± 3.6	26.3 ± 3.0	26.6 ± 3.7	0.197
**Body composition**					
Body fat (%)	33.8 ± 11.7	33.4 ± 7.5	34.0 ± 5.8	33.8 ± 8.2	0.978
Fat mass (kg)	26.36 ± 11.13	23.28 ± 6.50	24.47 ± 5.83	24.61 ± 7.84	0.519
LBM (kg)	49.92 ± 7.29	46.22 ± 9.05	47.66 ± 9.34	47.82 ± 8.67	0.459
ALM (kg)	22.28 ± 3.51	20.51 ± 4.76	22.11 ± 4.77	21.24 ± 4.42	0.501
**Strength/physical function**					
1RM leg press (kg)	129.9 ± 32.5	129.6 ± 56.1	129.4 ± 39.7	129.6 ± 43.6	0.999
1RM chest press (kg)	40.8 ± 16.8	39.4 ± 15.4	41.9 ± 16.0	40.7 ± 15.8	0.887
Hand-grip strength (kg)	31.9 ± 11.9	32.3 ± 11.7	31.7 ± 9.1	32.0 ± 10.6	0.984
Gait speed (m s^−1^)	1.97 ± 0.44	1.72 ± 0.35	1.96 ± 0.32	1.88 ± 0.38	0.077
Sit-to-stand (s)	10.64 ± 3.71	11.76 ± 2.32	10.85 ± 1.94	11.09 ± 2.67	0.422

*1RM, one-repetition maximum; ALM, appendicular lean mass; BMI, body mass index; LBM, lean body mass; M/F, male/female. P values are reported from one-way ANOVA between groups*.

### Assessments

The assessment procedure was identical in content and sequence at PRE and POST and performed over two consecutive days by the same personnel. These personnel were unblinded to the intervention groups due to these personnel also being involved in the execution of the exercise and/or dietary interventions. On day one, participants arrived to the laboratory after an overnight fast (> 8 h), having consumed 500 mL of water 2 h prior to their visit and engaged in minimal morning ambulation. After voiding of the bladder, body mass (to the nearest 0.1 kg) using a calibrated digital scales (SECA, Germany), height (to the nearest 0.01 m) using a wall-mounted stadiometer (Holtain, UK), and body composition by dual-energy X-ray absorptiometry (DXA; Lunar iDXA, GE Healthcare, USA) were measured. Regional measures of LBM of the upper and lower limbs (arms and legs, respectively) were obtained from the DXA scan analysis in order to calculate appendicular lean mass (ALM). Participants then consumed a small snack (cereal bar plus banana) and were allowed water ad libitum. Next, hand-grip strength of the dominant hand was measured to the nearest 0.5 kg using a hydraulic hand dynamometer (JAMAR, USA) (Roberts et al., 2011) followed by habitual gait speed (3 m), and five repetition sit-to-stand (Guralnik et al., 1994). On day two, participants reported to the exercise training facility (Medfit Proactive Healthcare, Dublin) for the assessment of lower and upper limb strength by one repetition maximum (1RM) on leg press and chest press machines, respectively (Milon, Germany). One week prior to the assessment at PRE, a familiarization session was performed. In this session, each of the tests described above were performed, and the correct lifting technique was demonstrated and practiced for each strength exercise, after which maximum strength was estimated using the multiple repetitions testing procedure. This estimate, in turn, informed the subsequent assessment of 1RM performed at PRE.

### Exercise Training Intervention

The exercise training intervention was fully supervised, small group (*n* = 4–6) training, and consisted of three exercise sessions per week (Monday, Wednesday, and Friday) of concurrent aerobic and resistance exercise training lasting ~40 min per session, which included a standardized warm-up and cool-down. The warm-up employed RAMP principles (R, raise heart rate and core/muscle temperature; A, activate musculature; M, mobilization of joints to create full range of motion; and P, potentiate/increase intensity in preparation for exercise protocol) over the course of 5 min including 3 min of low-to-moderate intensity aerobic exercise and 2 min of low intensity bodyweight movements/calisthenics. The cool-down was 5 min in duration consisting of low intensity bodyweight movements/calisthenics and walking to gradually lower heart rate, and incorporated static stretching of the major muscle groups of the upper and lower limbs. All training sessions were supervised and performed on the Milon Circle (Milon, Germany). Each session consisted 3 × 4 min intervals of aerobic exercise (Cross Trainer and Stationary Cycle Ergometer) and two rounds of the six resistance exercise circuit (Leg Press, Seated Row, Chest Press, Lat Pulldown, Leg Extension, and Tricep Dips). The aerobic and resistance exercises were interspersed by having participants complete three resistance exercises, followed by one 4 min interval of aerobic exercise, and repeating this pattern twice before concluding with three resistance exercises. A rest period of 30 s was taken in between each set of resistance exercise or interval of aerobic exercise.

For the aerobic exercise modes, the power output was adjusted to elicit a target intensity of 80% of age-predicted maximum heart rate for each 4 min interval throughout the training intervention in order to ensure that a progressive overload was continuously provided. For the resistance exercises, participants commenced training for weeks 1–4 with the prescription of 15 tempo-controlled repetitions of a given exercise in a 60 s period. The tempo for each 4 s repetition comprised of a 2 s eccentric movement, a 1 s pause, and a 1 s concentric movement and no pause between repetitions. For weeks 5–8, the prescription was adjusted to 12 tempo-controlled repetitions of a given exercise in a 60 s period. The tempo for each 5 s repetition comprised of a 3 s eccentric movement, a 1 s pause, and a 1 s concentric movement and no pause between repetitions. For weeks 9–12, the prescription was adjusted to 10 tempo-controlled repetitions of a given exercise in a 60 s period. The tempo for each 6 s repetition comprised of a 4 s eccentric movement, a 1 s pause, and a 1 s concentric movement and no pause between repetitions. Participants began the training intervention at ~60% of 1RM, but once an exercise could be completed comfortably for the 60 s period, an ~5% increment in weight to be lifted was added for the next training session in order to provide a progressive overload. For the weeks 5–8, and weeks 9–12, the load lifted was not prescribed based on %1RM but was manually adjusted by the practitioner according to the ability of each participant at the new prescription for repetitions/tempo, after which progressive overload was applied as described. The compliance with set duration and tempo was facilitated by the presence of a metronome and timer visible to participants on a digital display on each resistance training machine.

With 12 min of aerobic exercise and 12 min of resistance exercise, each training session, therefore, consisted of 24 min of active exercise, for a total of 72 min of active exercise each week (36 min aerobic exercise and 36 min resistance exercise). This exercise training program without dietary intervention has been previously shown by our group to elicit improvements in a range of measures of strength, physical function, and body composition in older adults (Timmons et al., 2018).

### Dietary Intervention

The dietary intervention targeted a high protein intake by providing meal and recipe suggestions using a whole food-based approach (i.e., powdered protein supplements and oral nutrition solutions) to achieve ~25–35 g (~0.4 g kg^−1^) of protein per meal. Each of these protein-rich meal recommendations also aimed to provide ~3 g of leucine. Participants from NUTR and NUTR+EX initially attended a briefing session in groups of 4–6 participants during which the dietary intervention was explained in detail. Participants were instructed to consume a protein-rich meal at breakfast, lunch, and dinner every day for the 12-week period, and in NUTR-EX, for one of these protein-rich meals to be within 60 min of each training session. Participants were asked to consume the specified portion in one sitting, and were asked not to split the portion over different eating occasions. Identical meal and recipe suggestions were provided to the participants in NUTR and NUTR+EX fortnightly by email for the duration of the study. These suggestions were informed by the USDA Food Composition Database, by taking food combinations and translating these into user-friendly portion sizes, meals, and recipes. Compliance with the dietary intervention was determined using a tick-box checklist completed per meal on a daily basis. Because of attendance at the supervised exercise sessions, contact with the NUTR+EX participants was weekly and informal, whereas contact with the NUTR participants was maintained formally with a fortnightly phone call to encourage participants to comply with the intervention. The EX group were asked to not to make any changes to their habitual dietary intake for the duration of the study. All participants completed a 3-day (two weekdays, one weekend day) portion-estimate food diary at PRE, week 6 (MID), and POST, which were analyzed using Nutritics Dietary Analysis Software (Nutritics, Ireland).

### Statistical Analysis

Data were evaluated using GraphPad Prism v8.4 (GraphPad Software, Inc., USA) and are presented as mean ± standard deviation (SD) at PRE and POST, and as mean difference (lower, upper 95% confidence limit of the mean difference) (95% CL) for data expressed as change from PRE. The data were tested for normality using the Shapiro-Wilk test prior to proceeding with the parametric tests described.

One-way analysis of variance (ANOVA) was performed to evaluate differences between groups at PRE for all parameters. Two-way (group × time) mixed ANOVA was performed to determine changes, if any, in response to intervention and differences, if any, between groups in those responses. When an interaction effect was indicated, between-group differences were evaluated using a one-way ANOVA performed on gain scores at POST with *post-hoc* comparisons performed with Tukey's correction applied, and for which multiplicity-adjusted *P* values are reported. Independent of the interaction effect, when a main effect of time was indicated, planned comparisons for within-group differences from PRE to POST were evaluated using *post-hoc* comparisons with Tukey's correction applied, and for which multiplicity-adjusted *P* values are reported. For all null hypothesis statistical testing, statistical significance was accepted at *P* < 0.05. Standardized differences in the mean were used to assess magnitudes of effects for between-group differences at POST, and for within-group changes from PRE to POST. These effect sizes were calculated using Cohen's *d* and interpreted using thresholds of *trivial* for <0.2, *small* for ≥ 0.2 to <0.5, *moderate* for ≥ 0.5 to <0.8, and *large* for ≥ 0.8.

## Results

### Compliance With Dietary and Exercise Training Interventions

There were no differences between groups at baseline for any parameter measured (Table 1). Attendance at the exercise training sessions averaged 87.4 ± 7.9% throughout the 12-week intervention, and did not differ by training group at 86.3 ± 10.2% and 88.7 ± 4.1%, for NUTR+EX and EX, respectively.

There was no change in dietary intake in EX throughout the intervention period whereas the dietary intervention was successful in increasing daily protein intake, and consequently daily energy intake, in NUTR and NUTR+EX (Table 2). Daily carbohydrate and fat intake did not differ between groups and remained similar over time (Table 2).

**Table 2:** Dietary macronutrient intakes during the 12 weeks of concurrent aerobic and resistance exercise training with or without dietary intervention for the respective groups.

		**Energy (kcal)**	**Carbohydrate (g)**	**Protein (g)**	**Protein (g kg^**−1**^)**	**Fat (g)**
NUTR	PRE	1,648 ± 441	173.6 ± 59.0	73.4 ± 25.7	0.99 ± 0.34	64.3 ± 22.3
	MID	1,949 ± 428^*^	154.6 ± 43.4	119.9 ± 30.5^**^	1.52 ± 0.45^**^	78.4 ± 23.9
	POST	1,989 ± 439^*^	168.5 ± 51.9	113.1 ± 29.3^**^	1.43 ± 0.39^*^	79.8 ± 27.1
EX	PRE	1,823 ± 344^#^	188.0 ± 38.1	80.0 ± 18.1	1.14 ± 0.35	69.6 ± 18.1
	MID	1,777 ± 437	175.9 ± 53.4	77.4 ± 18.1^#^^†^	1.10 ± 0.30	67.6 ± 23.4
	POST	1,793 ± 421	185.2 ± 63.7	75.6 ± 23.6^#^^†^	1.05 ± 0.28	66.6 ± 16.8
NUTR+EX	PRE	1466 ± 371	152.0 ± 50.7	65.8 ± 13.8	0.90 ± 0.20	56.2 ± 19.7
	MID	1,873 ± 449^*^	151.6 ± 43.6	117.8 ± 23.7^**^	1.59 ± 0.28^**^	73.4 ± 22.7
	POST	1,971 ± 837^*^	151.4 ± 51.2	117.1 ± 39.3^**^	1.57 ± 0.49^**^	70.6 ± 23.9

Data are mean ± SD. Statistical analysis was performed using two-way mixed ANOVA. Post-hoc pairwise comparisons with Tukey's correction were used to determine where differences existed between and within groups. Within-group differences compared to PRE are indicated by

* P < 0.05 and

** P < 0.01 for the annotated time point, and between-group differences are indicated by

# P < 0.05 for EX compared to NUTR+EX, and

† *P < 0.05 for EX compared to NUTR for the annotated time point. No between-group differences were observed between NUTR and NUTR-EX*.

### Strength Outcomes

**Figure 1:**
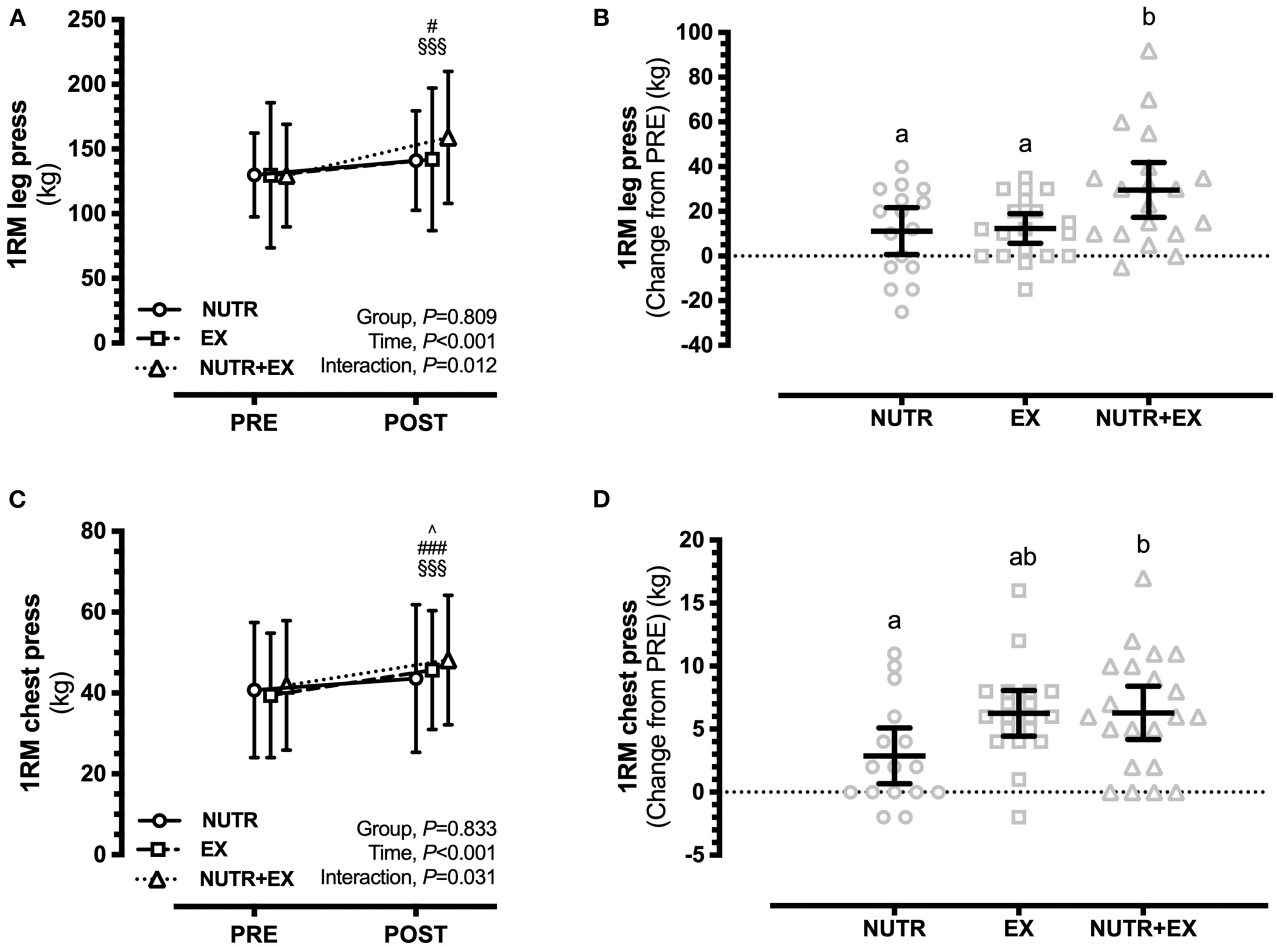
Changes in lower and upper limb muscle strength assessed by 1RM in response to 12 weeks of concurrent aerobic and resistance exercise training with or without dietary intervention. **(A)** Group mean ± SD for leg press; **(B)** Individual data points and mean difference with 95% CI for gain scores from PRE for leg press; **(C)** Group mean ± SD for chest press; **(D)** Individual data points and mean difference with 95% CI for gain scores from PRE for chest press. ^∧^ denotes significant difference from PRE to POST within NUTR; ^∧^*P* < 0.05; ^#^denotes significant difference from PRE to POST within EX; ^#^*P* < 0.05, ^*###*^*P* < 0.001; ^§^denotes significant difference from PRE to POST within NUTR+EX; ^§§§^ *P* < 0.001. Dissimilar letters demonstrate between-group differences (*P* < 0.05) in panels (**B,D**).

For the primary outcome, EX [12.3 (0.9, 23.8) kg; *P* = 0.031; *d* = 0.22] and NUTR+EX [29.6 (18.1, 41.0) kg; *P* < 0.001; *d* = 0.80] resulted in increases in 1RM leg press strength at POST (Time effect, *P* < 0.001), but a directional increase in NUTR [11.1 (−1.3, 23.6) kg] did not reach statistical significance (*P* = 0.093; *d* = 0.31) (Figure 1A). The increase in 1RM leg press strength in NUTR+EX was greater (interaction effect, *P* = 0.012) than the increases observed in NUTR by 18.5 (1.9, 35.0) kg (*P* = 0.026; *d* = 0.95), and in EX by 17.3 (1.4, 33.1) kg (*P* = 0.030; *d* = 0.70) (Figure 1B).

1RM chest press strength was increased (time effect, *P* < 0.001) in all groups, i.e., NUTR [2.9 (0.3, 5.5) kg; *P* = 0.026; *d* = 0.16], EX [6.3 (3.9, 8.7) kg; *P* < 0.001; *d* = 0.42], and NUTR+EX [6.3 (4.0, 8.6) kg; *P* < 0.001; *d* = 0.39] (Figure 1C). The increase in 1RM chest press strength in NUTR+EX was greater (interaction effect, *P* = 0.031) than the increases observed in NUTR by 3.4 (0.0, 6.8) kg (*P* = 0.026; *d* = 0.82), but the greater increase in EX by 3.3 (−0.1, 6.7) kg compared to NUTR did not reach statistical significance (*P* = 0.056; *d* = 0.92] (Figure 1D).

Hand-grip strength improved in all groups (time effect, *P* < 0.001), and no differences were observed between groups (interaction effect, *P* = 0.382) (Table 3).

**Table 3:** Changes from PRE to POST in body composition, strength, and physical function in response to the 12 weeks of concurrent aerobic and resistance exercise training with or without dietary intervention.

**‘**	**NUTR (*n* = 16)**	**EX (*n* = 19)**	**NUTR+EX (*n* = 21)**	**ANOVA *P* values**
**Body composition**				
Body mass (kg)	0.93 (0.12, 0.73)^*^	0.00 (−0.74, 0.74)	0.52 (−0.18, 1.23)	Time, *P* = 0.008 Group, *P* = 0.211 Interaction, *P* = 0.120
Fat mass (kg)	0.56 (−0.08, 1.19)	−0.26 (−0.84, 0.33)	−0.05 (−0.61, 0.50)	Time, *P* = 0.557 Group, *P* = 0.422 Interaction, *P* = 0.067
ALM (kg)	0.18 (−0.23, 0.59)	0.20 (−0.17, 0.58)	0.45 (0.09, 0.81)^**^	Time, *P* = 0.003 Group, *P* = 0.521 Interaction, *P* = 0.371
**Strength/physical function**				
Hand-grip strength (kg)	4.1 (2.1, 6.0)^***^	3.0 (1.2, 4.8)^***^	2.6 (0.9, 4.3)^**^	Time, *P* < 0.001 Group, *P* = 0.961 Interaction, *P* = 0.382
Gait speed (m s^−1^)	0.14 (−0.02, 0.31)	0.34 (0.19, 0.49)^***^	0.24 (0.09, 0.38)^***^	Time, *P* < 0.001 Group, *P* = 0.214 Interaction, *P* = 0.095
Sit-to-stand (s)	−2.51 (−3.52, −1.51)^***^	−3.00 (−3.95, −2.06)^***^	−2.87 (−3.74, −1.99)^***^	Time, *P* < 0.001 Group, *P* = 0.415 Interaction, *P* = 0.671

Data are reported as mean difference (95% CL). Statistical analysis was performed using two-way mixed ANOVA. For within-group differences, post-hoc comparisons with Tukey's correction were used to determine where differences existed compared to PRE as indicated by

* P < 0.05,

** P < 0.01, and

*** *P < 0.001 for the annotated time point*.

### Physical Function Outcomes

Gait speed improved in EX (*P* < 0.001; *d* = 1.01) and NUTR+EX (*P* < 0.001; *d* = 0.66) (time effect, *P* < 0.001), but a directional improvement in NUTR did not reach statistical significance (*P* = 0.105; *d* = 0.35) (Table 3). Sit-to-stand improved in all groups (time effect, *P* < 0.001), and no differences were observed between groups (interaction effect, *P* = 0.671) (Table 3).

### Body Mass and Body Composition Outcomes

**Figure 2:**
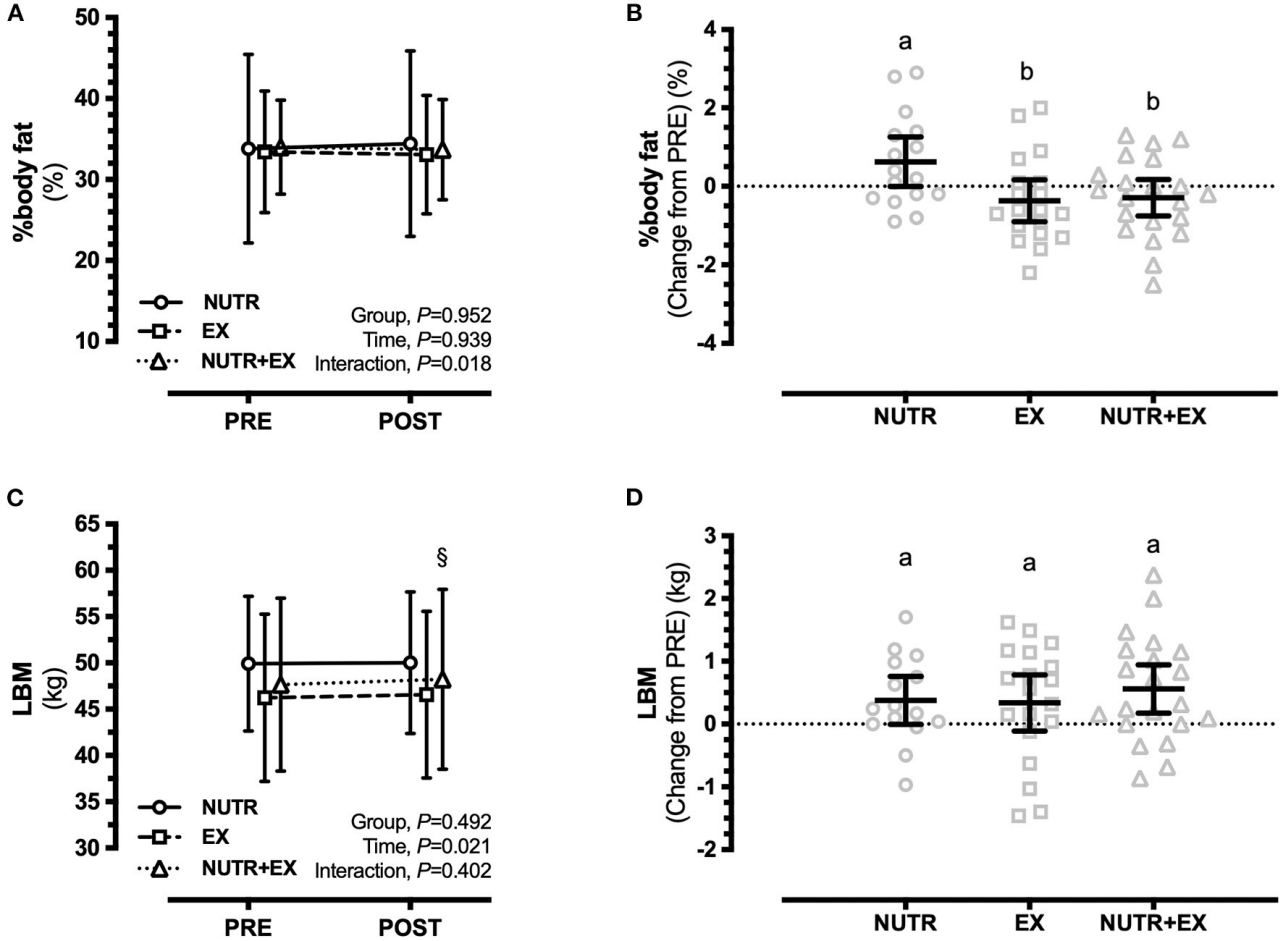
Changes in body composition assessed by DXA in response to 12 weeks of concurrent aerobic and resistance exercise training with or without dietary intervention. **(A)** Group mean ± SD for percentage body fat; **(B)** Individual data points and mean difference with 95%CI for gain scores from PRE for percentage body fat; **(C)** Group mean ± SD for LBM; **(D)** Individual data points and mean difference with 95% CI for gain scores from PRE for LBM. ^§^denotes significant difference from PRE to POST within NUTR+EX; ^§^*P* < 0.05. Dissimilar letters demonstrate between-group differences (*P* < 0.05) in panels **(B,D)**.

Body mass increased in NUTR [0.93 (0.12, 0.73) kg; *P* = 0.020; *d* = 0.11], but not in EX [0.00 (−0.74, 0.74) kg; *P* > 0.99; *d* = 0.00] or NUTR+EX [0.52 (−0.18, 1.23) kg; *P* = 0.202; *d* = 0.04] (interaction effect, *P* = 0.120; Table 3). Although the interaction effect (*P* = 0.067) for fat mass and the directional increase in fat mass in NUTR [0.56 (−0.08, 1.19) kg] did not reach statistical significance (*P* = 0.105; *d* = 0.05) (Table 3), an interaction effect was observed for percentage body fat (*P* = 0.018), with the increase in percentage body fat in NUTR being greater than changes observed in EX by 0.99 (0.10, 1.89)% (*P* = 0.027; *d* = 0.93) and in NUTR+EX by 0.92 (0.04, 1.79)% (*P* = 0.039; *d* = 0.94) (Figures 2A,B).

There was no interaction effect observed for LBM (interaction effect, *P* = 0.402) indicating the absence of between-group differences in LBM in response to the interventions (Figures 2C,D). Independent of the absence of between-group differences, within-group PRE-POST comparisons revealed LBM was increased in NUTR+EX [0.56 (0.01, 1.11) kg; *P* = 0.048; *d* = 0.06], but not in NUTR [0.09 (−0.54, 0.73) kg; *P* = 0.977; *d* = 0.01] or EX [0.34 (−0.24, 0.92) kg; *P* = 0.404; *d* = 0.04] (time effect, *P* = 0.021; Figures 2C,D). Similarly, ALM was increased in NUTR+EX [0.45 (0.09, 0.81) kg; *P* = 0.009; *d* = 0.09], but not in NUTR [0.18 (−0.23, 0.59) kg; *P* = 0.641; *d* = 0.05] or EX [0.20 (−0.17, 0.58) kg; *P* = 0.461; *d* = 0.04] (time effect, *P* = 0.003; Table 3).

## Discussion

This present study confirms the efficacy of concurrent aerobic and resistance exercise training to improve physical function in older adults (Wood et al., 2001; Karavirta et al., 2011; Timmons et al., 2018), with the addition of high protein whole food-based diet intervention augmenting some, but not all, of the training-induced outcomes. Most notably, the dietary intervention augmented training-induced increases in lower limb strength. In the absence of exercise training, this dietary pattern resulted in some improvements in physical function, but notably also resulted in an increase in percentage body fat.

The effect of protein supplementation in combination with resistance exercise on changes in strength, physical function, and LBM in older adults has been widely examined, with meta-analyses reporting conflicting conclusions in terms of positive (Cermak et al., 2012; Liao et al., 2017) or equivocal effects (Ten Haaf et al., 2018; Labata-Lezaun et al., 2020). Potential explanations for these discrepancies are divergent inclusion criteria for analyses, in particular, the inclusion of healthy and/or non-healthy, active, and/or ambulatory individuals and different age cut-offs. Generally, the potential to benefit from an exercise and/or dietary intervention is often greater for those who are least healthy, have low habitual physical activity, and/or inadequate protein intake. In the present cohort, baseline daily protein intake was similar to that previously reported in Irish older adults (Hone et al., 2020), and the dietary intervention successfully increased this intake from ~1.0 to ~1.5 g kg^−1^ d^−1^ in both NUTR and NUTR+EX. This increase was equivalent to ~40 to 55 g of additional protein per day. Moreover, daily energy intake was increased by ~21% in NUTR and ~34% in NUTR+EX. Dietary intervention alone (NUTR) resulted in improvements in several measures of physical function, i.e., chest press strength, hand-grip strength, and sit-to-stand. These outcomes are not unexpected as several studies of protein supplementation in the absence of exercise have demonstrated similar improvements in physical function in older adults (Bonnefoy et al., 2003; Tieland et al., 2012; Kim and Lee, 2013; Bauer et al., 2015; Chanet et al., 2017). However, the increase in body mass and percentage body fat in NUTR, albeit “trivial” in magnitude, may be considered a deleterious effect in the long-term if this results in adverse metabolic outcomes such as insulin resistance and/or anabolic resistance (Chang et al., 2012; Meex et al., 2019).

Concurrent aerobic and resistance exercise training is established as an efficacious strategy to improve strength, physical function, and body composition in middle-aged and older adults (Wood et al., 2001; Sigal et al., 2007; Davidson et al., 2009; Karavirta et al., 2011; Timmons et al., 2018). Similarly, in the present study, improvements in all strength (hand-grip strength, upper and lower limb strength) and functional (sit-to-stand and gait speed) outcomes were observed in both exercise training groups. However, the combination of both strategies (i.e., NUTR+EX) resulted in a markedly larger increase in lower limb strength. While between-group comparisons of change in LBM in response to the interventions did not reveal any differences between groups, the within-group analysis revealed that NUTR+EX was the only intervention that resulted in increases in LBM and ALM. The lack of change in LBM with concurrent aerobic and resistance training in the absence of dietary change (i.e., EX) is consistent with our previous study using this training regimen (Timmons et al., 2018). Indeed, meta-analyses of the effects of resistance exercise training alone suggests that a >1 kg increase in LBM would take >20 weeks of training three times per week (Peterson et al., 2011; Borde et al., 2015). In this regard, the increase in LBM in NUTR+EX is notable for being ~0.56 kg in 12 weeks, with ALM (~0.45 kg) accounting for the majority of this increase, notwithstanding that the magnitude of the effect size is “trivial.”

The markedly larger increase in lower limb strength in NUTR+EX (~25%) compared to other groups (NUTR, ~6%; EX, ~13%) is notable as an augmentation of the response to exercise training when additional dietary protein is consumed. This positive effect is, however, discordant with the conclusions of recent meta-analyses that conclude that providing additional protein does not augment improvements in strength after resistance exercise training in community-dwelling, non-frail older adults (Ten Haaf et al., 2018; Labata-Lezaun et al., 2020). There are some key methodological differences between the present study and studies included in these meta-analyses, including that the present study was comprised of concurrent aerobic and resistance exercise training. There is a large degree of heterogeneity in the various study designs, but broadly speaking, similar studies often provide additional protein only on training days (~3 days per week), or only achieve an increase of ~15–30 g of additional protein per day (Ten Haaf et al., 2018; Labata-Lezaun et al., 2020). Our dietary intervention therefore differs to many previous studies in that the quantity of additional protein per day was ~40 to 55 g, which was consumed on every day of week, and incorporated recent recommendations (Traylor et al., 2019) to provide an “even” distribution of protein throughout each day. These factors ultimately contributed to the average daily protein intakes reaching ~1.5 g kg^−1^, which is again greater than most previous studies in this domain (Ten Haaf et al., 2018; Labata-Lezaun et al., 2020). Notably, when a similar protein-enriched diet intervention using 2 × 80 g of cooked red meat 6 days per week was combined with thrice-weekly resistance exercise training for 16 weeks, leg extension strength increased by 28% in the meat plus exercise group as compared to 10% in the exercise only group (Daly et al., 2014). However, when the study was repeated with additional protein only on training days, no differences between groups was observed (Formica et al., 2020).

As for mechanisms by which additional dietary protein could augment the increase in strength in response to exercise training, the most likely explanation is a greater anabolic response both in the post-exercise period and generally on a per meal basis, together resulting in greater LBM accretion over time. While this was evident in the present study, it must again be acknowledged that the increase in LBM in NUTR-EX was “trivial” in magnitude, and was not significantly different between groups, so is unlikely to fully explain the differential effect observed on increased leg strength. That said, the present study assessed body composition *via* DXA, which is less sensitive for detecting small changes in LBM over time compared to magnetic resonance imaging (Delmonico et al., 2008; Tavoian et al., 2019), which is a more sensitive method for detection of change in muscle size by cross-sectional area (Cooper et al., 2013). Alternatively, it is widely acknowledged that changes in muscle strength are not strongly correlated with changes in muscle size or LBM, especially in older adults (Visser et al., 2000; Hughes et al., 2001; Delmonico et al., 2009), and factors other than change in tissue mass are likely to contribute to increases in muscle strength. One example from dietary intervention in older adults is the observation of a larger increase in leg strength per kg LBM when resistance exercise training was supplemented with cysteine-rich whey protein compared to casein protein (43.3 vs. 30.0% increase, respectively), yet in the absence of differences in change in LBM between groups (Karelis et al., 2015). Other contributors to an increase in strength independent of a change in muscle size or LBM could include alterations to neuromuscular action and/or muscle quality. Declines in these two aspects of skeletal muscle physiology have been proposed as central to the etiology of age-related muscle weakness in older adults that has been termed dynapenia, and for which resistance exercise training is an important countermeasure (Clark and Manini, 2010). Overall the present results for lower limb strength and LBM suggests that there are synergies between dietary intervention and exercise training that can be realized in adaptive outcomes, but this effect very much depends on the parameter of interest.

Given that many studies investigating higher dietary protein intake rely on powdered protein supplements and oral nutrition solutions (Cermak et al., 2012; Liao et al., 2017; Ten Haaf et al., 2018; Labata-Lezaun et al., 2020), the present study is novel in the approach to employ an exclusively whole food-based dietary co-intervention with concurrent aerobic and resistance exercise training. This approach is timely given recent calls for protein-based dietary interventions to focus on whole food sources (Burd et al., 2019; Marshall et al., 2020), in recognition of a potentially additive anabolic effect of whole foods over isolated sources of protein (Elliot et al., 2006; Burd et al., 2015; van Vliet et al., 2017; Abou Sawan et al., 2018). The present data, however, cannot suggest that whole foods are more efficacious than isolated sources, as direct comparison of such approaches would be required. Many factors contribute to reduction in energy and protein intake in older adults including a decrease in appetite with advancing age, the higher cost of more nutrient-dense foods, difficulty chewing fibrous foods, perceived food intolerances, and fear of eating excessive fat and cholesterol (Morley, 2005; Furman, 2006; Malafarina et al., 2013; Hung et al., 2019). In this context, 14% (3/21) of participants in the NUTR group failed to comply with the dietary intervention. Therefore, the translation of the present approach into other settings outside of a formal research trial would require cognizance of these issues, but clinicians should still consider emphasizing the significance of optimal daily protein intake when delivering advice around lifestyle change targeting skeletal muscle health.

The main limitation to the present study is the lack of a true, non-intervention control group, meaning that attributing within-group differences definitively to each intervention could be questioned. Factors such as the Hawthorne effect (McCambridge et al., 2014) resulting in generally better lifestyle habits during the intervention cannot be discounted, especially in NUTR, given that physical activity was not monitored in this study. In mitigation, our previous investigation in a similar cohort (age, sex, education, geographical location) employing many of the same assessments and the same duration of intervention observed no improvements in any of the measured outcomes in the non-intervention control group (Timmons et al., 2018). As the primary outcome was between-group differences in change in leg strength, the absence of a non-intervention control group does not impact the main conclusions from the present study. However, given that both energy intake and protein intake increased in NUTR+EX, it is not possible to attribute the benefits of the dietary intervention to protein specifically, or whether the general increase in energy availability supported the adaptive responses to exercise training. Lastly, one important caveat to this dietary approach is that it may only be appropriate for community-dwelling older adults given the challenges of nutrient provision in acute care settings and the myriad of factors influencing energy and protein intake in older adults (Morley, 2005; Furman, 2006; Malafarina et al., 2013; Hung et al., 2019). Caution may also be warranted in the case of excessive daily energy intake in the absence of exercise in this population.

## Conclusions

The present study is novel in its methodology in view of participants achieving high protein intakes (~1.5 g kg^−1^ d^−1^) exclusively through whole food sources, as opposed to supplementing with powdered protein supplements and oral nutrition solutions. While concurrent aerobic and resistance exercise training alone improved strength and physical function in older adults, combining an increase in dietary protein intake from whole foods with this type of exercise training was more advantageous for increasing lower limb strength and may support an increase in lean body mass, primarily in the form of appendicular lean mass. Such outcomes may be valuable in contexts such as during a period of rehabilitation after an adverse event that resulted in declines in muscle size and/or strength, or may inform clinicians and practitioners working in the field of exercise prescription, rehabilitation, and nutritional care of older adults.

## Data Availability Statement

The raw data supporting the conclusions of this article will be made available by the authors, without undue reservation.

## Ethics Statement

The studies involving human participants were reviewed and approved by University College Dublin Research Ethics Committee (permit: LS-17-22-Timmons-Egan) in accordance with the Declaration of Helsinki. The patients/participants provided their written informed consent to participate in this study.

## Author Contributions

JT and BE designed the study. JT, MH, and KC performed the assessments. JT, MH, and OD were responsible for performing the interventions. JT, MH, and BE analyzed the data. JT, MH, and BE wrote the first draft of the manuscript. KC and BE edited the manuscript. BE had the primary responsibility for final content. All authors contributed to editing and approved the final version of the manuscript.

## Conflict of Interest

BE is formerly (previous 36 months) or presently funded under the Health, and Glycemic Management pillars, respectively, of the Enterprise Ireland-funded technology centres Meat Technology Ireland, and Food for Health Ireland, whose focus is on the potential benefits of meat and dairy ingredients for human health. The remaining authors declare that the research was conducted in the absence of any commercial or financial relationships that could be construed as a potential conflict of interest.
