# Supplementary material for: Increased Leg Strength After Concurrent Aerobic and Resistance Exercise Training in Older Adults Is Augmented by a Whole Food-Based High Protein Diet Intervention
Source: Front Sports Act Living. 2021 Mar 26;3:653962. doi: 10.3389/fspor.2021.653962 (PMC8034230; doi:10.3389/fspor.2021.653962)
Supplement: Supplementary file 1 [file Table_1.DOCX]

Supplementary Material

Timmons JF, Hone M, Cogan KE, Duffy O and Egan B (2021) Increased Leg Strength After Concurrent Aerobic and Resistance Exercise Training in Older Adults Is Augmented by a Whole Food-Based High Protein Diet Intervention. Front. Sports Act. Living 3:653962. doi: 10.3389/fspor.2021.653962

**Supplementary Figure 1.** CONSORT flow chart for study participants
